# Nintedanib Ameliorates Bleomycin-Induced Pulmonary Fibrosis, Inflammation, Apoptosis, and Oxidative Stress by Modulating PI3K/Akt/mTOR Pathway in Mice

**DOI:** 10.1007/s10753-023-01825-2

**Published:** 2023-05-09

**Authors:** Lin Pan, Yiju Cheng, Wenting Yang, Xiao Wu, Honglan Zhu, Meigui Hu, Yuquan Zhang, Menglin Zhang

**Affiliations:** 1 Department of Respiratory and Critical Care Medicine, The Affiliated Hospital of Guizhou Medical University, Guiyang, 550004 China; 2 Department of Respiratory and Critical Care Medicine, Guiyang First People’s Hospital, Guiyang, 550004 China; 3 Guizhou Medical University, Guiyang, 550004 China

**Keywords:** Pulmonary fibrosis, Nintedanib, Bleomycin, Oxidative stress, Apoptosis, PI3K/Akt/mTOR pathway.

## Abstract

Idiopathic pulmonary fibrosis (IPF) seriously threatens human life and health, and no curative therapy is available at present. Nintedanib is the first agent approved by the US Food and Drug Administration (FDA) in order to treat IPF; however, its mechanism of inhibition of IPF is still elusive. According to recent studies, nintedanib is a potent inhibitor. It can antagonize platelet-derived growth factor (PDGF), basic fibroblast growth factor (b-FGF), vascular endothelial growth factor (VEGF), etc*.*, to inhibit pulmonary fibrosis. Whether there are other signaling pathways involved in IPF remains unknown. This study focused on investigating the therapeutic efficacy of nintedanib in bleomycin-mediated pulmonary fibrosis (PF) mice through PI3K/Akt/mTOR pathway. Following the induction of pulmonary fibrosis in C57 mice through bleomycin (BLM) administration, the mice were randomized into five groups: (1) the normal control group, (2) the BLM model control group, (3) the low-dose Nintedanib administration model group, (4) the medium-dose nintedanib administration model group, and (5) the high-dose nintedanib administration model group. For lung tissues, morphological changes were found by HE staining and Masson staining, ELISA method was used to detect inflammatory factors, alkaline water method to estimate collagen content, and western blotting for protein levels. TUNEL staining and immunofluorescence methods were used to analyze the effect of nintedanib on lung tissue and the impacts and underlying mechanisms of bleomycin-induced pulmonary fibrosis. After 28 days, bleomycin-treated mice developed significant pulmonary fibrosis. Relative to bleomycin-treated mice, nintedanib-treated mice had markedly reduced degrees of PF. In addition, nintedanib showed lung-protective effects by up-regulating antioxidant levels, down-regulating inflammatory protein expression, and reducing collagen accumulation. We demonstrated that nintedanib ameliorated bleomycin-induced lung injury by inhibiting the P13K/Akt/mTOR pathway as well as apoptosis. In addition, significant improvement in pulmonary fibrosis was seen after nintedanib (30/60/120 mg/kg body weight/day) treatment through a dose-dependent way. Histopathological results further corroborated the effect of nintedanib treatment on remarkably attenuating bleomycin-mediated mouse lung injury. According to our findings, nintedanib restores the antioxidant system, suppresses pro-inflammatory factors, and inhibits apoptosis. Nintedanib can reduce bleomycin-induced inflammation by downregulating PI3K/Akt/mTOR pathway, PF, and oxidative stress (OS).

**Supplementary Information:**

The online version contains supplementary material available at 10.1007/s10753-023-01825-2.

## INTRODUCTION

The most normal form of chronic pulmonary fibrosis (PF) refers to idiopathic pulmonary fibrosis (IPF), with the poor prognosis compared to numerous malignant tumors [1]. Recently, IPF pathogenesis and treatment have received a lot of attention worldwide. The etiology of IPF is not well understood. However, various factors, such as an imbalance between anti-fibrotic mediators, pro-fibrotic mediators supporting ECM expansion, fibroblast growth, differentiation and recruitment, and tissue remodeling, are considered important factors for IPF [2]. Oxidative stress (OS), epigenetic alterations, cell aging inflammation, apoptosis, and telomere shortening have been identified as contributing factors to aging and related pulmonary diseases [3]. Since the lungs show extremely high susceptibility to OS because of its specific anatomy, location, and function, OS has been considered a key molecular mechanism during PF development [4–7]. Multiple pieces of evidence have proved a critical effect of OS on fibrosis and a positive association between OS levels in the body and PF degree [8]. Therefore, anti-oxidative is suggested as the potential therapeutic target in the inhibition of pulmonary fibrosis. The US FDA approved pirfenidone and nintedanib on October 15, 2014, as two drugs that can be utilized for the treatment of IPF. They have been demonstrated to prolong the lifespan of patients, improve lung function, reduce the frequency of hospitalization, enhance the quality of life, and decelerate the rate of disease progression [9]. Pirfenidone belongs to the pyridinone class of drugs, with multiple mechanisms of action, including anti-pulmonary fibrosis, anti-inflammatory, and antioxidant properties. Studies have revealed that pirfenidone can impede pulmonary fibrosis by interfering with the signaling pathways of fibroblast growth factor (FGF) and platelet-derived growth factor (PDGF) *in vivo*. On the other hand, nintedanib can slow down the progression of idiopathic pulmonary fibrosis and reduce the rate of lung function decline. These drugs have distinct mechanisms of action and are recommended as first-line therapeutic options for treating idiopathic pulmonary fibrosis, according to clinical guidelines [10].Multiple researches have shown that nintedanib serves as an intracellular inhibitor against several tyrosine kinases [11]. It can effectively alleviate the IPF disease progression, thus bringing in a new era of IPF treatment. Nintedanib inhibits vascular endothelial growth factor receptors (VEGFRs), fibroblast growth factor receptors (FGFRs), as well as platelet-derived growth factor receptors (PGFRs), all of which are related to IPF pathogenesis [12]. In primary human embryonic lung fibroblasts collected from fibrotic cases, nintedanib has been shown to hinder the pro-fibrotic impacts of FGH and VEGF and reduction in TGF-β-induced collagen deposition [13]. The possibility of other signaling pathways being inhibited by nintedanib in IPF still remains and requires further research.

PI3K/AKT/mTOR pathway has the important impact on modulating cell growth, autophagy, apoptosis, energy metabolism, and protein biosynthesis [14, 15]. Recent researches have shown that the pathway is strongly related to lung fibrosis and its activation and insufficient autocracy promoted PF occurrence [16–18]. Therefore, a potential approach to treating PF may be blocking the PI3K/AKT/mTOR pathway.

However, the function of nintedanib on the P13K/Akt/mTOR pathway in regulating pulmonary fibrosis remains unknown. As a result, the present work explores the effect and mechanism of nintedanib. The bleomycin-mediated pulmonary fibrosis model was adopted for analyzing the therapeutic impacts of nintedanib and evaluating whether or not its inhibitory effects on pulmonary fibrosis are through suppressing PI3K/Akt/mTOR pathway.

## MATERIAL AND METHODS

### Reagents

Bleomycin and nintedanib were offered by MCE and Shanghai McLean Biochemical Technology Co., Ltd., respectively. For Western blot analysis, ɑ-SMA (1:1000, Cat. No. 19245S), PI3K (1:1000, Cat. No. 4257S), Akt (1:1000, Cat. No. 2983S), p-Akt (1:2000, Cat. No. 4060 T), Cleaved Caspase-3 (1:1000, Cat..No. 9664S), and mTOR (1:1000, Cat. No. 5536 T) were purchased from CST, whereas GAPDH (1:10,000, Cat. no. ab181602), p-PI3K (1:1000, Cat. no. ab182651), and ColIII (1:1000, Cat. no. ab184993) were purchased from Abcam. Tumor necrosis factor ɑ (TNF-ɑ), interleukin-1β (IL-1β), and interleukin-6 (IL-6) ELISA kits were offered by Beijing Sizhengbai Biotechnology Co., Ltd. (Beijing, China). Reactive oxygen species (ROS), glutathione peroxidase (GSH-Px), malondialdehyde (MDA), and superoxide dismutase (SOD) were provided by Nanjing Jiancheng Bioengineering Institute.

### Animals

The approval of the experiments was obtained by the Institutional Animal Care and Use Committee of Guizhou Medical University. Male C57BL/6 mice (16–20 g) within 6–7 weeks were offered by the Laboratory Animal Research Center of Guizhou Medical University (Guizhou, China). We housed mice in cages at 23 ± 2 °C and 60 ± 10% humidity with the 12 h/12 h light/dark cycle. Mice had free access to food and water. Following the 7-day observation, mice were randomized into five groups (*n* = 12), including control, model, low-, medium-, and high-dose nintedanib groups (30, 60, and 120 mg/kg body weight per day). In treated mice, PF was triggered by intratracheal bleomycin instillation once at 5 mg/kg, while controls received instillation of equivalent normal saline. Seven days following bleomycin treatment, mice in treatment groups were given nintedanib by gavage, while the control and bleomycin groups could receive the same amount of normal saline for 21 days. Body weight (BW) was measured every day with the purpose of detecting growth changes. On day 28, each mouse was given an injection of 1% sodium pentobarbital for anesthesia, followed by the collection of blood, lung tissues, and bronchoalveolar lavage fluid (BALF) for further experiments.

Then, mice were euthanized with an anesthesia overdose.

### Model Protocol and Atreatment Scheme

For this study, 60 male C57 BL/6 mice were randomly divided into five groups using a random number table method: Sham operation group, model group, low-dose nintedanib group (30 mg/kg body weight per day), medium-dose nintedanib group (60 mg/kg body weight per day), and high-dose nintedanib group (120 mg/kg body weight per day), with 12 mice in each group. The mice were anesthetized using intraperitoneal injection of 1% pentobarbital sodium, fixed in a supine position with an oral opening device to facilitate the injection of bleomycin. To enable clear observation of the injection process, the tongue of the mouse was pulled out and the tongue abdomen was pressed with a tongue depressor. Endotracheal intubation was immediately performed as soon as the mouse inhaled. The model group and the drug group were slowly injected with Bleomycin (BLM) at a dose of 5 mg/kg, respectively, and the drug solution was evenly distributed in the lungs by rotating the mice. In contrast, the sham group was injected with the same amount of normal saline. Following natural awakening, the mice were allowed to drink and eat freely. On the 7th day post-modeling, the mice were intragastrically administered nintedanib and randomly divided into five groups based on body weight using a random block design: (1) sham operation group (control), (2) model control group (BLM), (3) low-dose nintedanib group (30 mg/kg body weight per day), (4) medium-dose nintedanib group (60 mg/kg body weight per day), and (5) high-dose nintedanib group (120 mg/kg body weight per day). In (3), (4), and (5) groups, nintedanib was administered intragastrically at the specified dosage, while the model and sham operation groups were given an equivalent amount of normal saline via intragastric administration. The drug was administered once daily for 28 days.

### Histopathology

Lung tissues were subjected to a 24-h 4% paraformaldehyde (PFA) fixation, followed by a 2-h 70% PFA, overnight 80% PFA, 2-h 90% PFA, and 100% PFA treatment twice per hour under ambient temperature. Ethanol dehydration. The paraffin-embedded tissues were sliced into 4-µm sections, which were later stained with hematoxylin and eosin (HE) and Masson’s trichrome in line with specific protocols. Sections were found using a microscope, and images were captured, followed by PF severity assessment in accordance with the description provided by Szapiel et al. [19].

### Immunohistochemical Analysis of Type I Collagen

Tissue blocks measuring < 0.5 × 0.5 × 0.1 cm were collected and rinsed five times using PBS. Tissue blocks were analyzed by immunohistochemical assays according to specific instructions. Sections were then fixed, embedded, sectioned, and deparaffinized, followed by 20 min repairing of anti-fibrotic solutions using 0.01 M sodium citrate. Followed by this, 3% hydrogen peroxide was supplemented to quench endogenous catalase activity for 10 min. The tissue was then left at 37 °C for 20 min in order to eliminate nonspecific binding. Afterward, each section was washed, followed by overnight incubation under the condition of 4 °C with type I collagen antibody (ab270993). Thereafter, sections were nurtured with biotinylated rabbit secondary antibody (ab6728) and left at 37 °C for 20 min. Afterward, the sections were incubated with HRP-conjugated streptomycin ovalbumin working solution at 37 °C for a period of 20 min, followed by routine dehydration, tissue clear, diaminobenzidine staining, and sealing with neutral resin.

### Hyp Level Detection

The frozen lung tissue (approximately 30 mg) was sliced into pieces and ground with a homogenizer at a low temperature. Hydroxyproline content was then analyzed by adopting a hydroxyproline kit (Cat. No. A030–2–1, Nanjing Jiancheng Bioengineering Institute) based on the manufacturer’s protocol. Afterward, lung tissue was subjected to hydrolysis at 100 °C for 20 min, and accordingly, pH was adjusted. Later, the resultant mixed sample was subject to incubation at 60 °C for a quarter, cooling, and centrifugation at 3500 rpm for 10 min at room temperature. Absorbance (OD) values were measured at 550 nm for each sample. Data was determined as µg of hydroxyproline per mg of wet lung weight.

### Enzyme-Linked Immunosorbent Assay (ELISA)

BALF was gathered and centrifuged at 2000 rpm for a period of 10 min at the apartment temperature, followed by preservation at − 80 °C. IL-1β, IL-6, and TNF-ɑ levels were decided with ELISA reagents based on IL-1β (Cat. No. CME0015), IL-6 (Cat. No. CME0006), and TNF-ɑ (Cat. No. CME0004), in accordance with the protocol of the manufacturer (Beijing Sizhengbai Biotechnology Co., Ltd.).

### Western Blotting (WB) Analysis

Lung tissue was homogenized in RIPA lysis buffer (Beijing Solarbio Science & Technology Co., Ltd.). Additionally, BCA protein assay kit was adopted for measuring the total protein levels of a sample (Beijing Solarbio Science & Technology Co., Ltd.). After quantification with the BCA kit, proteins (20 µg) were separated with the use of the 10% concentration of SDS-PAGE and transferred onto PVDF membranes. Blocking was done using 5% milk for 1 h at the apartment temperature. Subsequently, overnight primary antibody incubation was carried out at 4 °C. The applied antibodies were ɑ-SMA, COL1A1, TGF-β,mTOR, p-mTOR, Akt, p-Akt, PI3K, p-PI3K, Cleaved Caspase-3, and GAPDH (loading control). Membranes were rinsed thrice by TBS including 0.1% Tween 20 (TBST), followed by 1 h incubation using HRP-labeled anti-rabbit antibody (Cat: CST) at room temperature. After rinsing with TBS five times, bands were determined using an enhanced chemiluminometer and Image Lab software (Bio-Rad). Meanwhile, Image J software was used to analyze protein gray values. Protein levels were determined based on GAPDH. Samples derive from the same experiment and that blots were processed in parallel.

### MDA Level, SOD, GSH-px, and ROS Activity Determination

ROS, MDA, GSH-PX, and SOD contents were evaluated using different detection kits as per the manufacturer’s protocol. ROS levels within lung tissue were measured by ROS measurement kit (directory number E004–1–1 Nanjing) and the probe through DCFH-DA (2,7-dichlorofluorescein Diacetate) on the basis of the instruction of the manufacturer. Absorbance was measured at 530 nm, where its fluorescent strength was proportional to the active oxygen level.

GSH-Px kit (directory number: A005–1, Nanjing built into the Institute of Biological Engineering) was employed to analyze the activity of GSH-Px according to the manufacturer’s protocol, which measures restored glutathione use. Inhale light at 412 nm and horizontal level of glutathione peroxidase is defined as µ/mL.

MDA (directory number: A003–1, Nanjing Institute of Biological Engineering Research Institute) was used to determine the MDA level of the lung tissue as per the manufacturer’s protocol. For detecting MDA concentration in the lung tissue, sulfuric acid degradation products were measured at the maximal wavelength of 532 nm. The light absorption values were compared with the calibration curve plotted according to the MDA standard. The results are represented by nmol/mL.

SOD vitality within lung tissue was analyzed by SOD measurement kit (directory number: A001–3, Nanjing to build a biological engineering research institute) in accordance with the instruction of the manufacturer. Meanwhile, the suction light was determined under the wavelength of 450 nm. The data is represented by U.

### TUNEL Staining of Lung Tissues

After dewaxing and hydration of paraffin-embedded sections, they were treated with a proteinase K working solution and a TUNEL reaction mix. Later, 50 mU/L transformation pods were spread on the dried samples. The mixture was covered with a coverslip or sealing film, followed by 30 min reaction at 37 °C within a dark box. Finally, 50–100 mU/L methyl green and DAB were introduced into the resultant mixture. A fluorescence microscope was used to observe sections.

### Statistical Analysis

In order to perform data analysis, GraphPad Prism and SPSS 22.0 were employed. Every assay was conducted in triplicates, and results were represented as mean ± SD. The unpaired *t*-test was conducted with the aim of comparing two groups, whereas ANOVA plus Tukey’s test was adopted for comparing several groups. *P* < 0.05 was thought to be of statistical significance.

## RESULTS

**Fig. 1:**
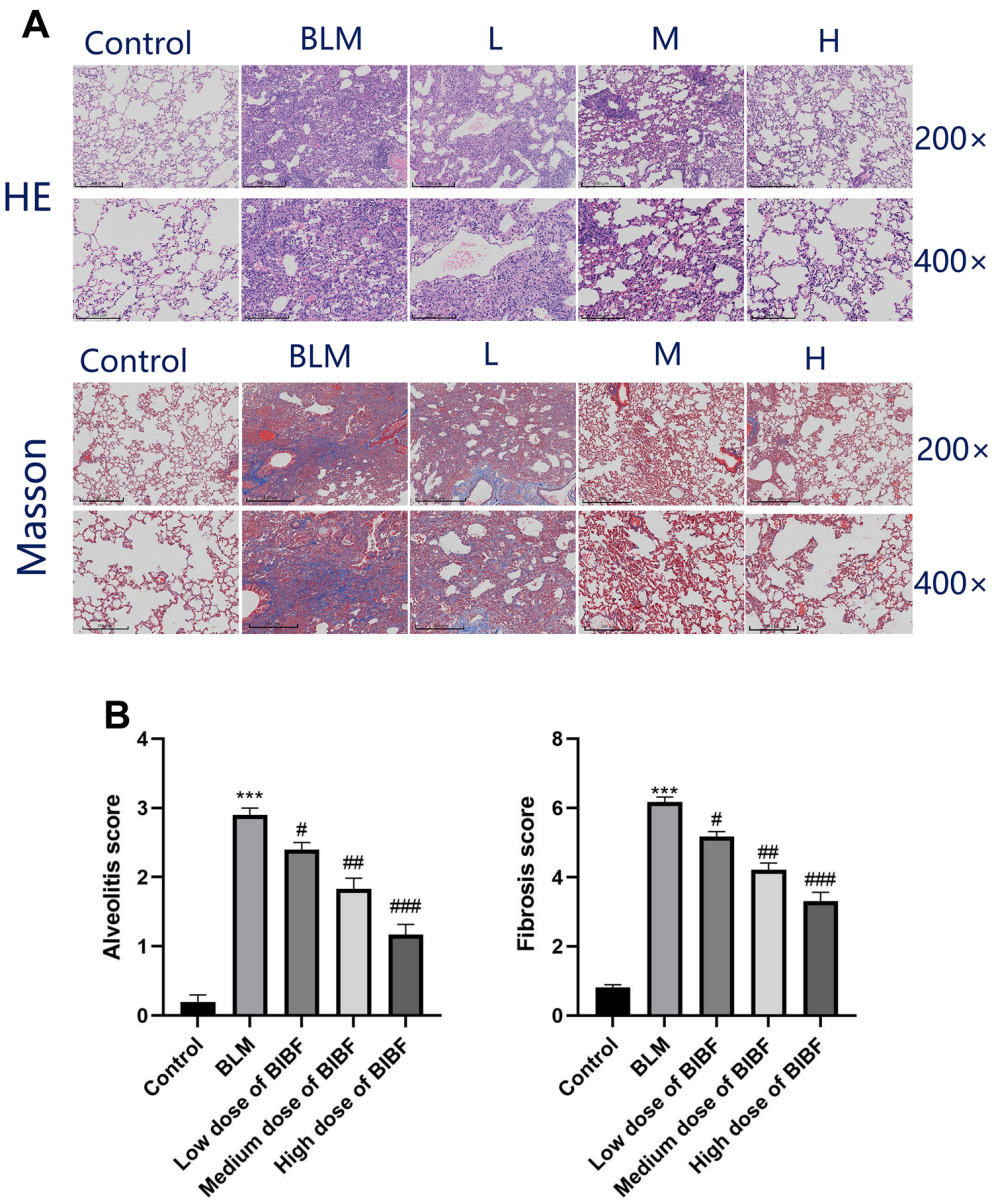
Nintedanib attenuates bleomycin-mediated mice PF. **A** Changes in lung tissue morphology were analyzed by HE and Masson staining at magnifications × 200 and × 400. **B** Statistical analysis of alveolitis and PF scores, relative to control group, ***P < 0.0005, relative to model group, #*P* < 0.05, ##*P* < 0.005, ###*P* < 0.0005.

To explore the impact of nintedanib on bleomycin-mediated PF, pathological alterations of lung tissue were observed by HE and Masson staining in each group. HE staining was used to determine inflammation, while collagen deposition within different sections was analyzed by Masson’s trichrome staining. After bleomycin administration, a remarkable increase in alveolar septa thickening, as well as collagen deposition within lung tissue, was observed (Fig. 1A). However, nintedanib treatment has a reverse effect, with a gradual decrease in the alveolitis and PF degrees as the dose of nintedanib increased relative to the bleomycin group. Szapiel’s semiquantitative approach was utilized to grade alveolitis and PF degrees (Fig. 1B). This study evaluated fibrosis using a scoring system. A score of 0 indicated normal lung tissue, while a score of 1 indicated mild thickening of alveolar or bronchial walls. A score of 3 indicated moderate thickening of alveolar or bronchial walls without significant damage to alveolar structure, while 5 points indicated the presence of cord fibrous bands or small areas of fibrous foci, with evident damage to alveolar structure. A score of 7 indicated severe deformation of alveolar structure and extensive fibrous foci formation, leading to “honeycomb lung.” Finally, a score of 8 indicated diffuse fibrosis throughout the lung tissue, corresponding to the severity of lesions between scores 2, 4, and 6. The scores were used to assess the severity of fibrosis in the lungs. When compared with the control group, the bleomycin group had markedly aggravated alveolitis and PF degrees (*P* < 0.05). Nonetheless, when compared with the bleomycin group, nintedanib treatment significantly (*P* < 0.05) reduced alveolitis and pulmonary fibrosis, which gradually improved with increasing nintedanib dose.

**Fig. 2:**
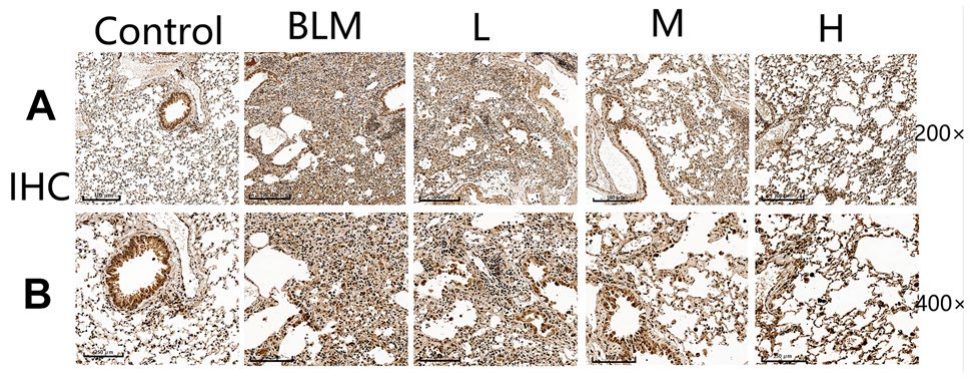
Nintedanib improves bleomycin-mediated PF. **A** Representative images showing IHC staining for collagen I within lung tissues, at original magnification × 200. **B** IHC showing collagen I within lung tissues. Representative images of staining, original magnification × 400.

An enhancement in type I collagen proportion represents a hallmark of PF occurrence. To investigate the effect of nintedanib on PF, we checked type I collagen levels in lung tissue through immunofluorescence. Four weeks after administration, type I collagen levels were detected in lung tissue, and the levels markedly increased within the bleomycin group in comparison with the control group, corroborating with the immunofluorescence results. Nintedanib administration significantly reduced type I collagen levels, as notable differences were shown between the model and all groups, except for the low-dose nintedanib group (Fig. 2).

**Fig. 3:**
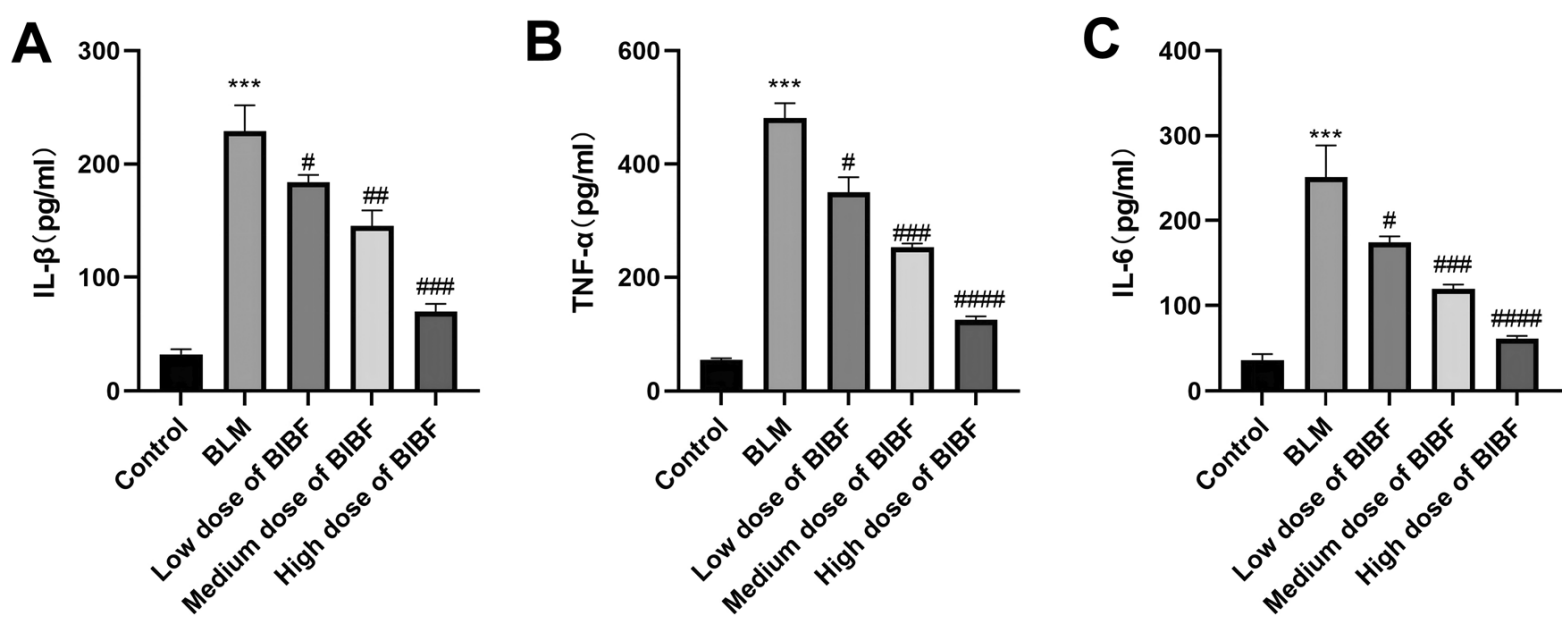
Nintedanib can reduce inflammation in lung tissues from PF mice. Inflammatory factors were detected by the ELISA kit. **A** IL-1β, **B** TNF-α, and **C** IL-6 levels within BALF of diverse groups. relative to control group, ***P < 0.0005, relative to model group, #*P* < 0.05, ###*P* < 0.005, ####*P* < 0.00005.

IL-1β, IL-6, and TNF-α within BALF in normal control rats and bleomycin-induced PF mice were measured to investigate the inflammatory activity. After bleomycin treatment, pro-inflammatory factor influx was significantly elevated. A notable enhancement (*P* < 0.05) in IL-1β, IL-6, and TNF-ɑ expression was mediated by bleomycin. Whereas increasing doses of nintedanib inhibited the expression of the above three inflammatory markers. This suggested that nintedanib has good anti-inflammatory properties. Nintedanib effectively suppressed bleomycin-mediated inflammation, as evident by significant differences (*P* < 0.05) in the model group in comparison with other groups (Fig. 3).

**Fig. 4:**
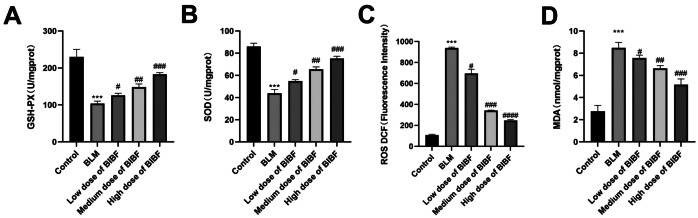
Nintedanib decreases OS damage caused by bleomycin. Effects of nintedanib at different doses on **A** GSH-Px, **B** SOD, **C** ROS, and **D** MDA levels in lung tissue. Results are obtained by ≥ 3 independent assays, relative to control group, ***P < 0.0005, relative to model group, #*P* < 0.05, ##*P* < 0.005, ###*P* < 0.0005.

In addition, the effect of nintedanib on OS was measured by analyzing ROS, MDA, SOD, and GSH-PX contents in lung tissue from the mouse model. Based on the obtained results, compared to the control group, ROS and MDA contents notably added in the bleomycin group, while SOD and GSH-PX activities notably lowered. However, after nintedanib administration, SOD and GSH-PX activities remarkably enhanced, whereas MDA and ROS levels gradually declined in a dose-dependent manner. The obtained findings suggest that nintedanib could improve oxidative stress induced by bleomycin (Fig. 4).

**Fig. 5:**
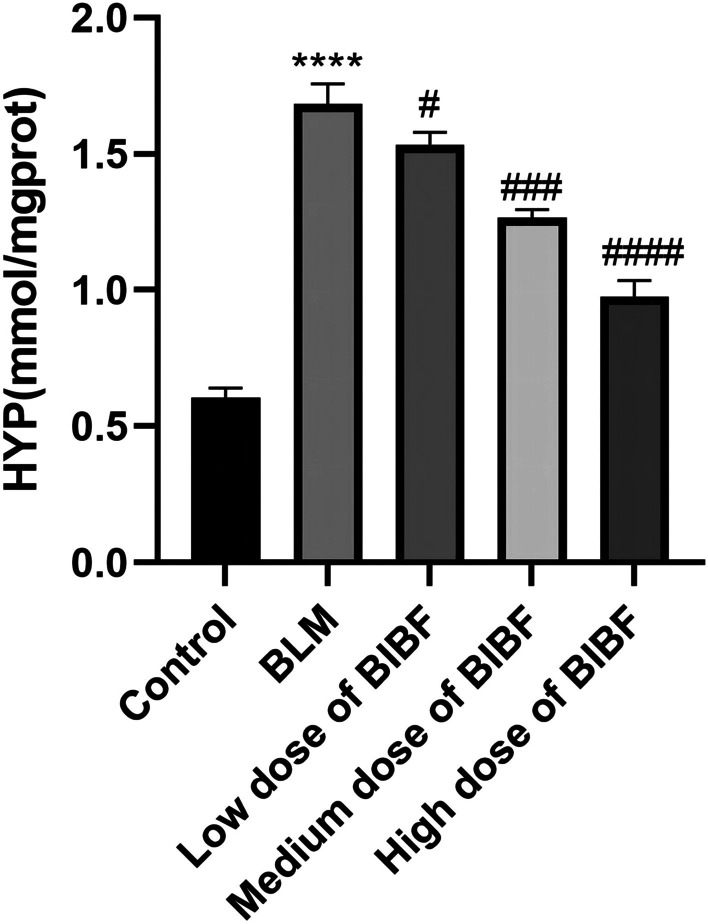
Effect of nintedanib on the collagen content in bleomycin-induced mice lung tissue. Changes in HYP expression after nintedanib treatment, relative to control group, ****P < 0.00005, relative to model group, #*P* < 0.05, ###*P* < 0.0005, ####*P* < 0.00005.

Bleomycin treatment induced collagen deposition of hydroxyproline within lung tissue and significantly increased the HYP content. However, nintedanib prevented collagen deposition through a dose-dependent manner, suggestive of the great therapeutic efficacy of the highest dose (Fig. 5).

**Fig. 6:**
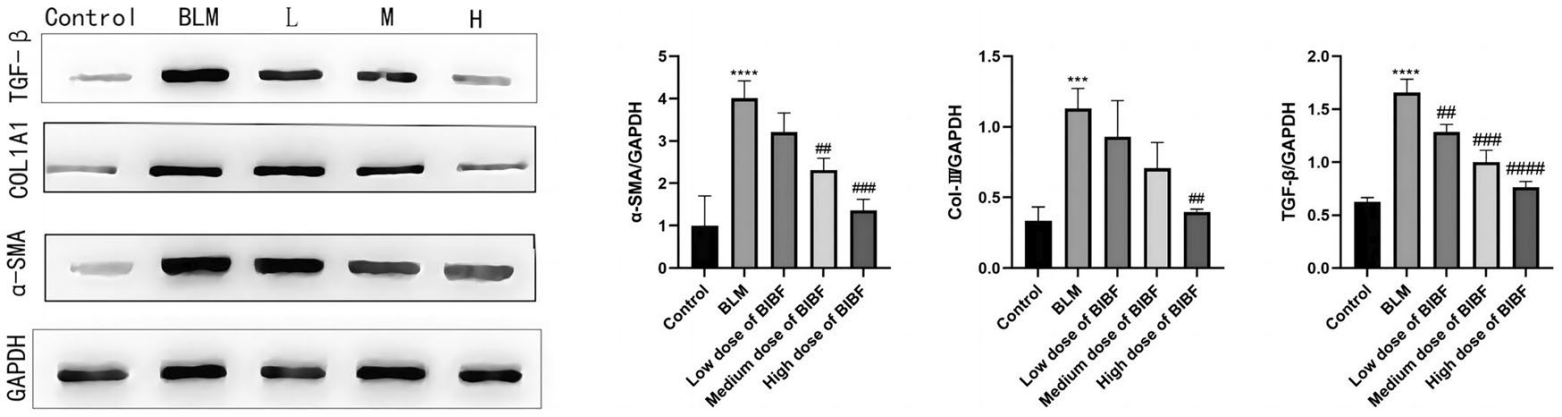
Effect of nintedanib on fibrosis-associated protein expression within the lung tissue of mice. The ColIII and ɑ-SMA protein expression within lung tissue of each group of mice were measured by western blotting. relative to control group, ***P < 0.0005, relative to model group, ##*P* < 0.005, ###*P* < 0.0005.

The impact of nintedanib on fibrosis-associated protein levels within lung tissue from bleomycin-mediated mice was assessed. The western blotting results from lung tissues of each group confirmed the effect of nintedanib on alleviating bleomycin-mediated PF. ColIII, ɑ-SMA, and TGF-β protein expression within lung tissues from the bleomycin group notably increased (*P* < 0.05) (Fig. 6). Nevertheless, the protein expression significantly declined through a nintedanib dose-dependent manner.

**Fig. 7:**
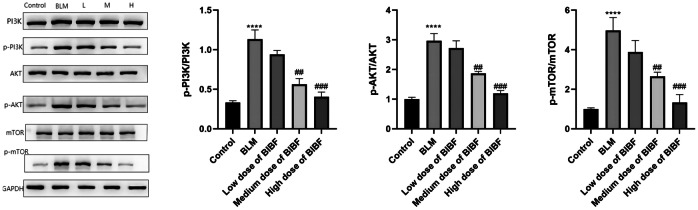
Impact of nintedanib on PI3K/Akt/mTOR pathway-associated protein levels within lung tissue. PI3K, p-PI3K, Akt, p-Akt, mTOR, and p-mTOR protein expression within lung tissues from mice were measured using western blotting, relative to control group, ****P < 0.00005, relative to model group, ##*P* < 0.005, ###*P* < 0.0005.

PI3K/Akt/mTOR pathway serves as the key intracellular signaling pathway associated with cell proliferation, migration, apoptosis, survival, autophagy, protein biosynthesis, and reverse transcription. Additionally, inhibiting PI3K/Akt/mTOR pathway enhances autophagy while improving PF. p-PI3K, p-Akt, and p-mTOR protein levels of the bleomycin group notably enhanced (*P* < 0.05) compared to the normal group. However, nintedanib treatment significantly decreased the expression through the dose-dependent manner. Additionally, the results also revealed a dose-dependent downregulation of PI3K/Akt/mTOR pathway phosphorylation levels in the nintedanib treatment group compared with the bleomycin group. These above findings indicated that nintedanib could inhibit the PI3K/Akt/mTOR pathway phosphorylation (Fig. 7).

**Fig. 8:**
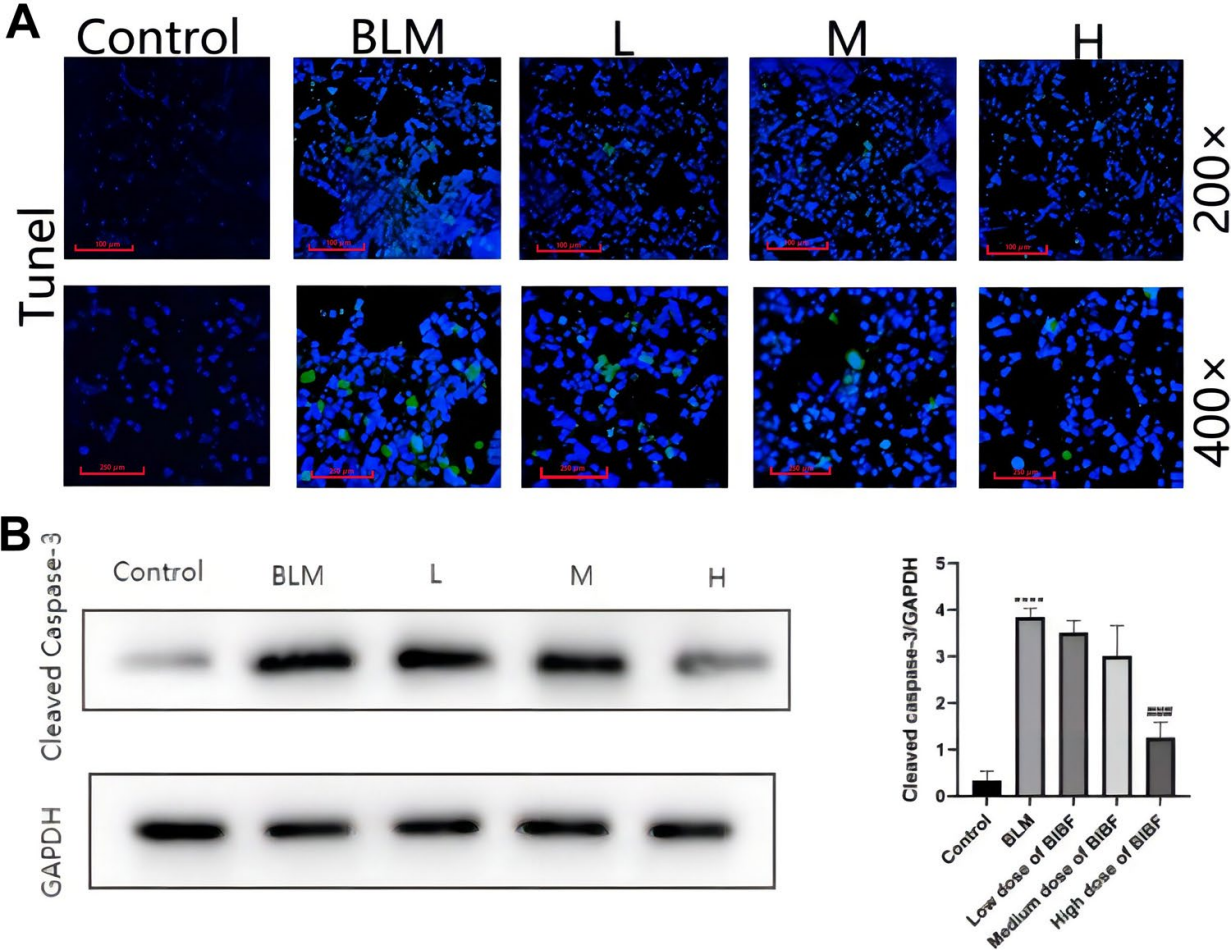
Nintedanib inhibits the changes of apoptosis protein expression within mouse lung tissues experiencing bleomycin-mediated PF. **A** TUNEL staining, **B** nintedanib reduces bleomycin-induced Cleaved Caspase-3 expression. relative to control group, ****P < 0.00005, relative to model group, ###*P* < 0.0005.

During PF development, a slight increase in apoptotic cell number reflects the PF pathogenicity. This study explored the involvement of nintedanib in the apoptosis of lung tissue by TUNEL staining that identifies tissue apoptosis. TUNEL staining analysis showed that relative to the normal group, the apoptotic cell number in the model group showed significant increase. However, after nintedanib administration, apoptotic cells gradually decreased in a dose-dependent way in comparison with the bleomycin group. The maximum reduction was seen in the group with nintedanib high dose. In addition, pro-caspase-3 cleavage and activation were achieved by initiating caspases (namely, caspases 2, 8, 9, 10, 11, and 12) to form caspase-3. In response to apoptotic stimuli, the bleomycin group enhanced the cleaved caspase-3 protein level, which was suppressed via nintedanib administration (Fig. 8).

## DISCUSSION

IPF represents the chronic progressive condition featured by an abnormal accumulation of fibrotic tissue in the lung parenchyma, resulting in decreased life quality and lower life expectancy [20]. The pathogenesis of IPF is not well understood, highlighting the need for more research in the domain in order to develop new specific therapeutic drugs according to the disease pathogenesis. The bleomycin-mediated PF animal model has been widely utilized to study IPF [21]. Previous studies have shown that clinically safe nintedanib can slow down PF development and delay lung function decline, meanwhile improving patient’s life quality [22, 23]. In a research carried out by Boxhamme et al*.*, they found that pretreatment of patients with nintedanib (an inhibitor of receptor tyrosine kinase) downregulated ROS and TGM-2 and showed potential antioxidation and immunomodulation effects in treating chronic dysfunction post lung transplantation [24]. In another study by Fois et al*.*, they found that nintedanib exerted benefits for OS and inflammation markers among IPF cases [25].

Based on these, the current work analyzed the mechanism of action of nintedanib in PF. We found that nintedanib significantly improved BLM-mediated pulmonary apoptosis and PF, mitigated OS and inflammatory responses, and partially via the PI3K/Akt/mTOR pathway.

BLM induces pulmonary inflammation and PF within a short time, while BLM administered intratracheally up-regulates fibrotic factor levels, like IL-1β, IL-6, TNF-α, and TGF-β, etc. [21]. In this study, we observed pathological changes caused by inflammation in lung tissue. Following BLM treatment, Hyp content and inflammatory cells significantly increased, and the alveolar septa were significantly thickened, as observed by HE and Masson staining. Surprisingly, nintedanib treatment reduced collagen deposition and infiltration of inflammatory cells within mouse lungs. TGF-β is a key factor in the cytokine network of pulmonary fibrosis. It has the ability to attract monocyte macrophages and neutrophils, enhance the expression of cytokines related to pulmonary fibrosis, and expedite the fibrosis process. The results also showed that nintedanib could attenuate bleomycin-mediated mouse PF and inflammation. ɑ-SMA, type III collagen, and TGF-β were reduced after nintedanib treatment. ɑ-SMA is a marker indicating the activation of fibroblasts, and its level causes fibroblast transformation into myofibroblast [26]. Also, a notable reduction in TNF-ɑ, IL-1β, and IL-6 levels was observed.

OS has been reported to facilitate fibrosis of various organs [27, 28]. Excess reactive oxygen species may break down cellular macromolecules, leading to tissue damage and fibrosis [29] and an imbalance in ROS production [30, 31]. Therefore, in this study, we evaluated OS by determining ROS, glutathione peroxidase, malondialdehyde, and superoxide dismutase activities. We found that MDA and ROS expression significantly increased, while GSH-Px and SOD expression significantly decreased in the BLM group. Interestingly, nintedanib could reverse these effects and significantly decrease the levels of MDA and ROS while increasing SOD and GSH-Px expression. This suggested that nintedanib could significantly inhibit the oxidative stress injury induced by bleomycin and thus improve pulmonary fibrosis.

PI3K/Akt/mTOR pathway is a leading pathway that mediates cell survival by inhibiting apoptosis and stimulating cell proliferation [32, 33]. The pathway is becoming a therapeutic target for IPF [34]. Various researches have shown that as PI3K and mTOR A class I isoform inhibitor, HEC68498 shows compelling properties with high selectivity. It exhibits potent protection from inflammation and fibrosis and provides excellent therapeutic effects even at low effective doses [35]. In the present study, nintedanib resulted in decreased p-PI3K/PI3K, p-mTOR/mTOR, and p-Akt/Akt and sum ratios, indicating that nintedanib can inhibit bleomycin-induced activation of PI3K/Akt/mTOR pathway. Apart from that, Akt activates its downstream mTOR pathway to keep low autophagic activity in fibroblasts, which in turn retains high proliferation and low apoptosis rates [36]. This work explored the detailed mechanism underlying nintedanib-mediated PF. Morphological observations showed that nintedanib decreased TUNEL-positive cell number. Besides, nintedanib also reversed the BLM-associated Cleaved Caspase-3 up-regulation, suggesting that nintedanib has a protective effect on lung tissue apoptosis via PI3K/Akt/mTOR pathway.

Therefore, from all these findings, we infer that nintedanib protects against bleomycin-mediated PF via modulating PI3K/Akt/mTOR pathway.

The current study makes significant contributions to the field. It is the first study to systematically investigate the relationship between the anti-pulmonary fibrosis mechanism of nintedanib and the PI3K/Akt/mTOR signaling pathway. The results confirmed that nintedanib can alleviate pulmonary fibrosis by regulating the PI3K/Akt/mTOR signaling pathway. The study also aimed to provide insights into the molecular signaling pathway of nintedanib against pulmonary fibrosis and to identify potential clinical treatments for IPF. However, the study has some limitations, including the lack of *in vitro* cell studies, which require further investigation.

## Conclusion

To conclude, although nintedanib can decrease PF in clinical practice, the related mechanism remains largely unclear. The present work demonstrated the potential of nintedanib in restoring the antioxidant system meanwhile inhibiting inflammatory factors and apoptosis. Histopathology results showed that nintedanib significantly ameliorated bleomycin-induced lung injury. In addition, this work, for the first time, provides evidence that nintedanib improved oxidative stress, inhibited inflammatory response, and improved lung tissue apoptosis by downregulating PI3K/Akt/mTOR signaling pathway, ultimately improving lung fibrosis. The results shed new light on nintedanib’s protection against PF and its related mechanism, which serves as a rationale for nintedanib for IPF treatment.

## Data and Material Availability

All data utilized in this work could be obtained from the corresponding author upon request.

## Supplementary Information

Below is the link to the electronic supplementary material.

Supplementary file1 (PDF 14349 KB)
