# Supplementary material for: Nintedanib Ameliorates Bleomycin-Induced Pulmonary Fibrosis, Inflammation, Apoptosis, and Oxidative Stress by Modulating PI3K/Akt/mTOR Pathway in Mice
Source: Inflammation. 2023 May 9;46(4):1531–42. doi: 10.1007/s10753-023-01825-2 (PMC10359208; doi:10.1007/s10753-023-01825-2)

Figure 6：GAPDH

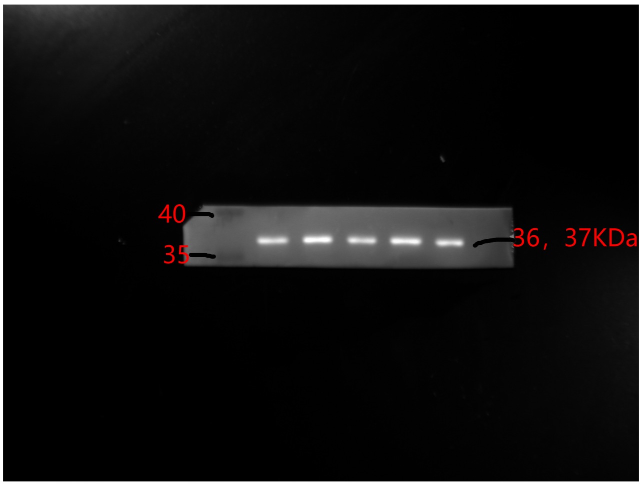

Figure 6：α-SMA

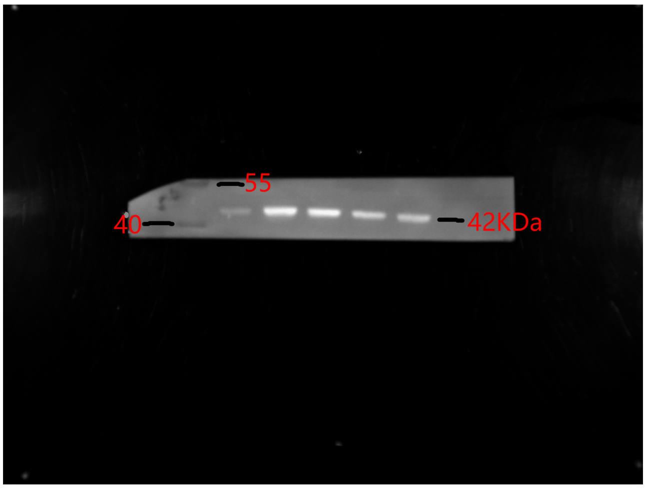

Figure 6：Col-Ⅲ

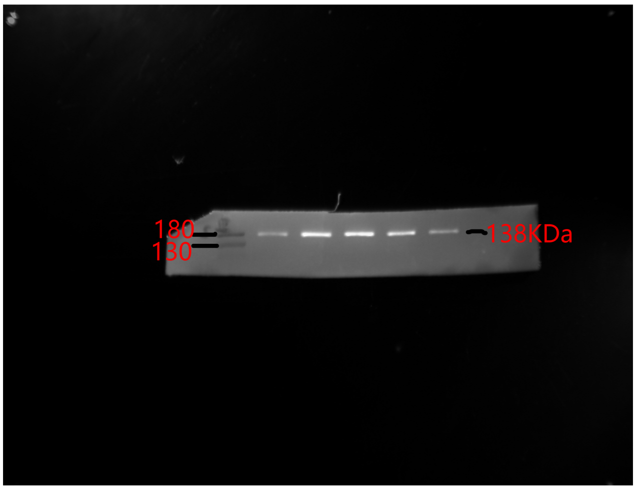

The original image of Figure 6, the pores of each band are Control, Model, nintedanib 30mg/kg, nintedanib 60mg/kg， nintedanib 120mg/kg from left to right.The samples derive from the same e xperiment and the same time andthat gels/blots were processed in parallel.Because the experimen t consisted of 5groups

Figure8:GAPDH

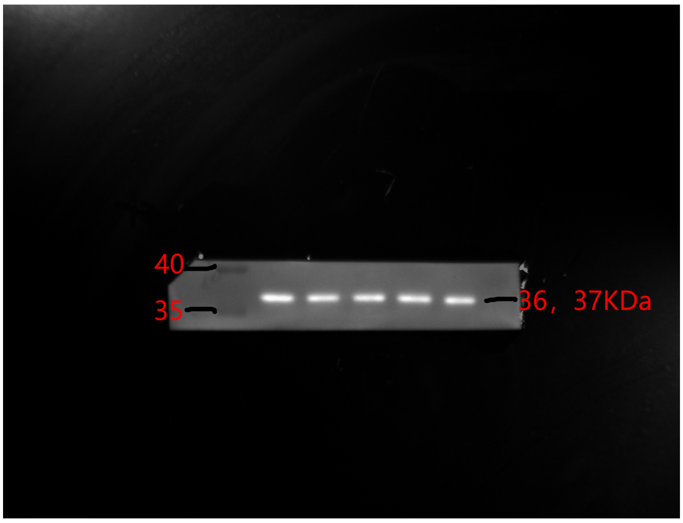

Figure8:Cleaved caspase-3

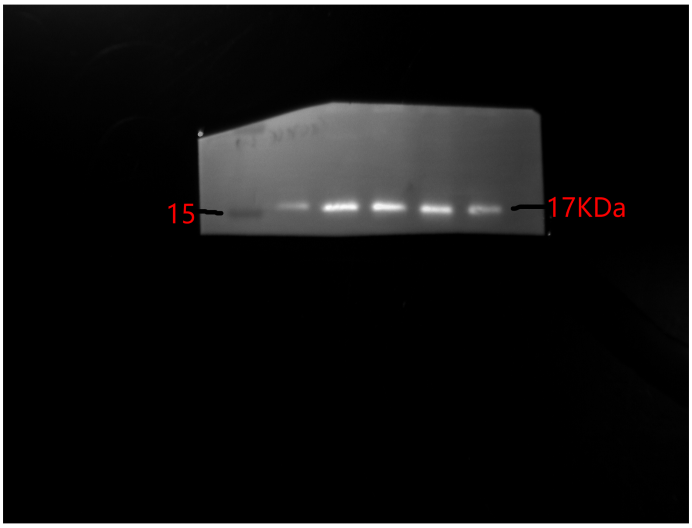

Figure 7：GAPDH

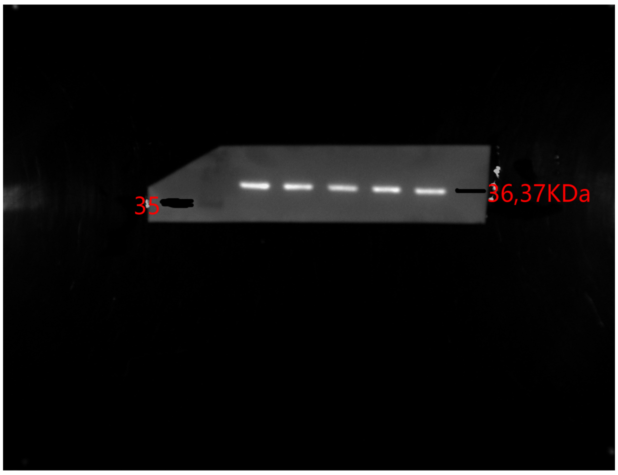

The original image of Figure 6, the pores of each band are Control, Model, nintedanib 30mg/kg,nintedanib 60mg/kg， nintedanib 120mg/kg from left to right.The samples derive from the same experiment and the same time andthat gels/blots were processed in parallel.Because the experiment consisted of 5groups

Figure 7：PI3K

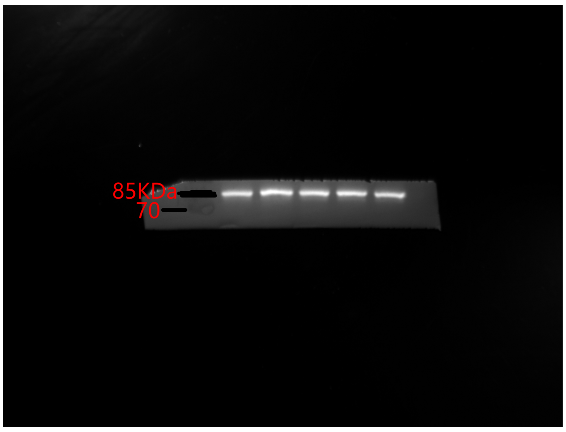

Figure 7：p-PI3K

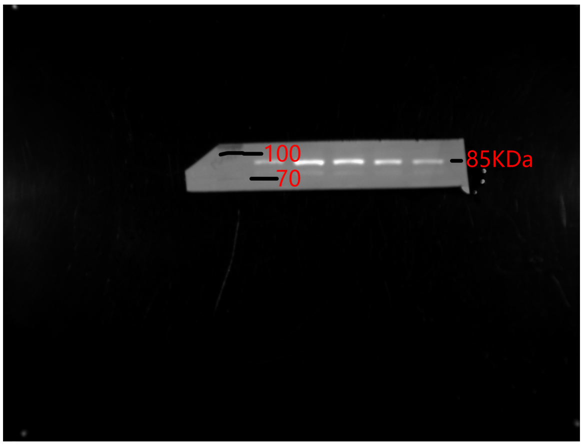

Figure 7：AKT

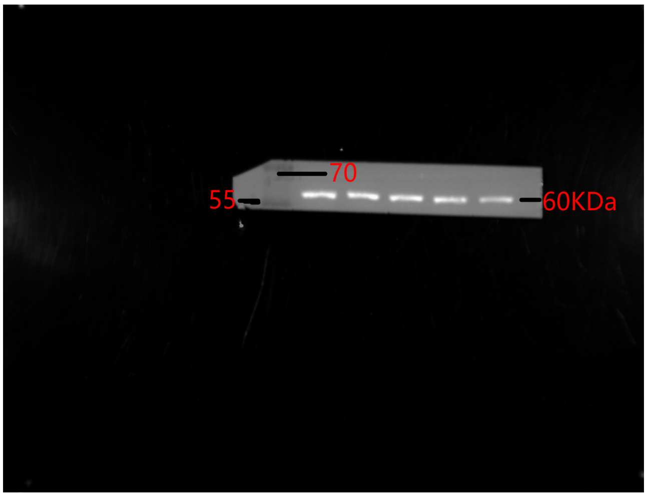

Figure 7：p-AKT

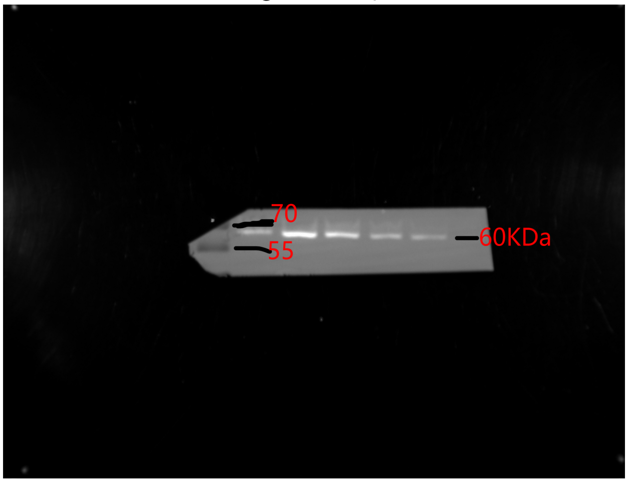

Figure 7：MTOR

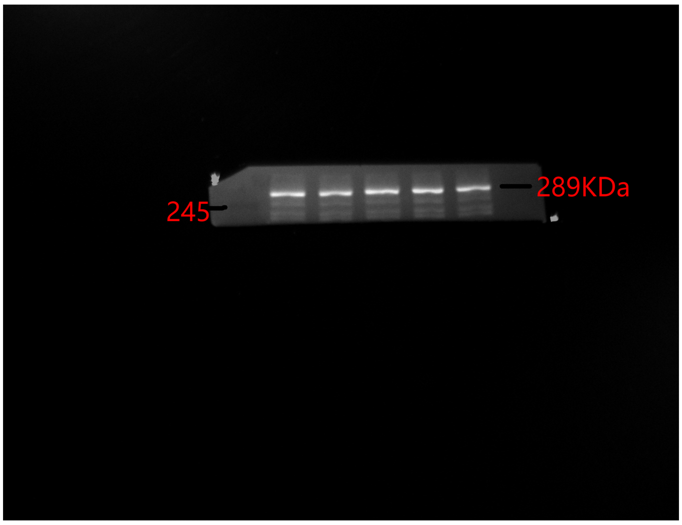

Figure 7：p-MTOR

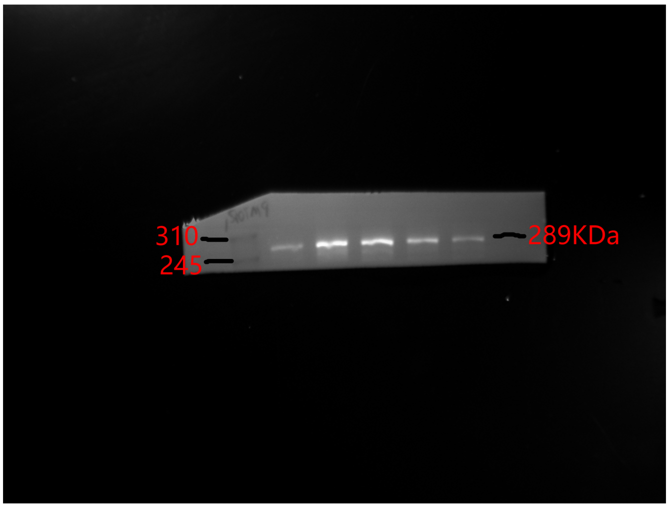

Supplement: Supplementary file 1 — Supplementary file1 (PDF 14349 KB) [file 10753_2023_1825_MOESM1_ESM.pdf]
